# Supplementary material for: Cross-species transcriptomics reveals bifurcation point during the arterial-to-hemogenic transition
Source: Commun Biol. 2023 Aug 9;6:827. doi: 10.1038/s42003-023-05190-6 (PMC10412572; doi:10.1038/s42003-023-05190-6)
Supplement: Supplementary file 2 — Supplementary information [file 42003_2023_5190_MOESM2_ESM.pdf]

# Cross-species Transcriptomics Reveals Bifurcation Point During the Arterial-to-Hemogenic Transition

Shaokang Mo[1,2,3†], Kengyuan Qu[2,3†], Junfeng Huang[2,3†*], Qiwei Li[2,3], Wenqing Zhang[1*], Kuangyu Yen[2,3*]

[1]Division of Cell, Developmental and Integrative Biology, School of Medicine, South China University of Technology, Guangzhou, China

[2] State Key Laboratory of Experimental Hematology, National Clinical Research Center for Blood Diseases, Haihe Laboratory of Cell Ecosystem, Institute of Hematology & Blood Diseases Hospital, Chinese Academy of Medical Sciences & Peking Union Medical College, Tianjin 300020, China

[3] Tianjin Institutes of Health Science, Tianjin 301600, China

† These authors contributed equally

* To whom correspondence should be addressed:

Kuangyu Yen, PhD

E-mail: kuangyuyen@ihcams.ac.cn

Junfeng Huang, PhD

E-mail: jfhuang.dg@gmail.com

Wenqing Zhang, PhD

E-mail: mczhangwq@scut.edu.cn

## Supplementary information includes following items:

## Supplementary Figure 1-9:

**Supplementary Figure 1.** Comparison of mouse and human midbrain using HomologySeeker

**Supplementary Figure 2.** Construction of EHT ensembles

**Supplementary Figure 3.** Identification of Homologous-HVGs in EHT ensembles

**Supplementary Figure 4.** Query projection without mouse IAC in the reference

**Supplementary Figure 5.** Unsupervised clustering of human EHT ensembles

**Supplementary Figure 6.** Human C6 subcluster is characterized by pre-HE signatures

**Supplementary Figure 7.** Integration of GJA5+ AECs into the human EHT ensemble

**Supplementary Figure 8.** Differential expression analysis between pre-HE/early AEC and HE/late AEC

**Supplementary Figure 9.** Identification of signaling pathways mediated by ligand modules

**Supplementary Figure 10.** Gating strategy for flow cytometry analysis used in Figure 4

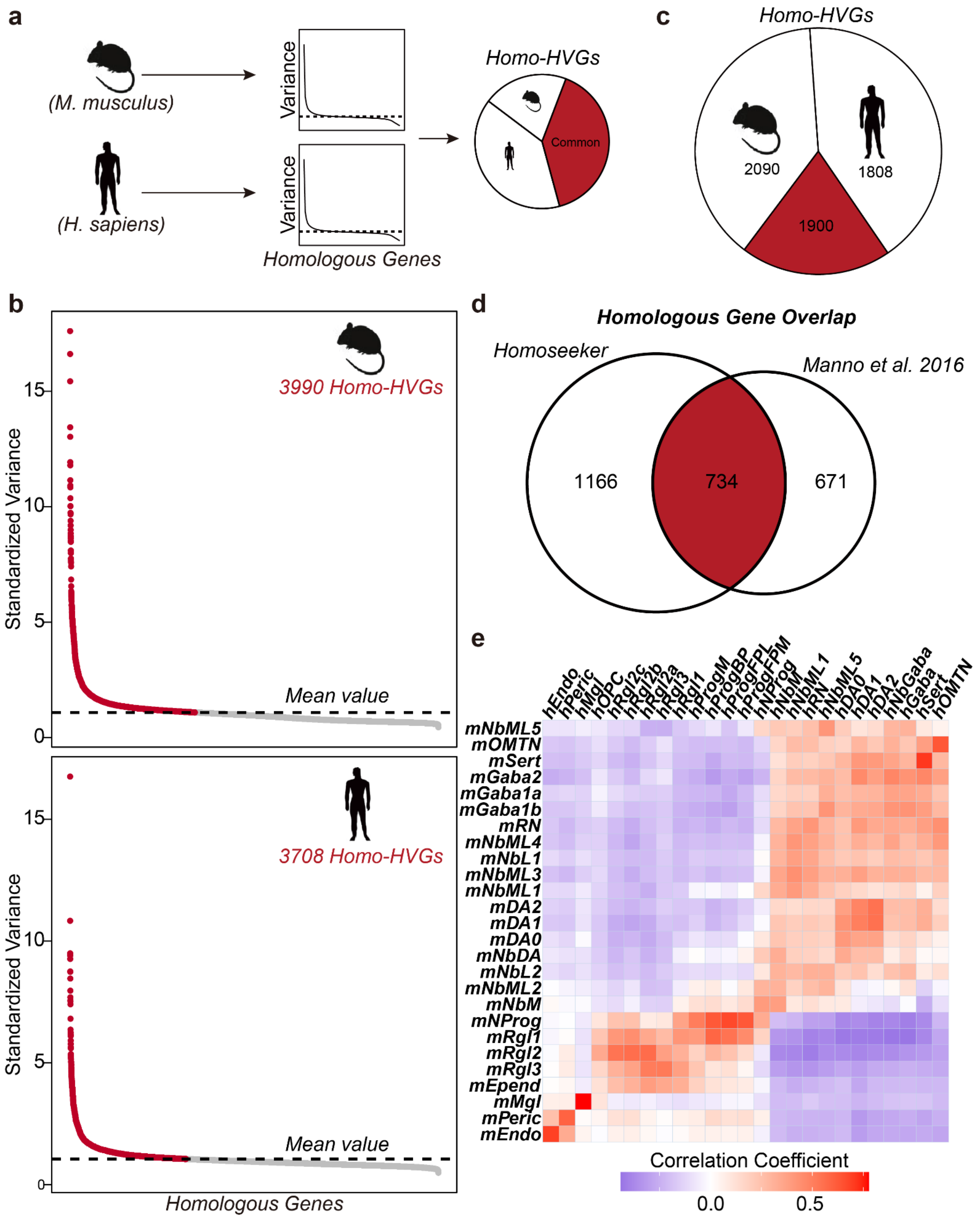
a
(M. musculus)
(H. sapiens)
Variance
Variance
Homologous Genes
Homo-HVGs
Common
c
Homo-HVGs
2090
1808
1900
b
3990 Homo-HVGs
Standardized Variance
Mean value
3708 Homo-HVGs
Standardized Variance
Mean value
Homologous Genes
d
Homologous Gene Overlap
Homoseeker
Manno et al. 2016
1166
734
671
e
hEndo
hPeric
hMgl
hOPC
hRgl2c
hRgl2b
hRgl2a
hRgl3
hRgl1
hProgM
hProgBP
hProgFPL
hProgFPM
hNProg
hNbM
hNbML1
hRN
hNbML5
hDA0
hDA1
hDA2
hNbGaba
hGaba
hSert
hOMTN
mNbML5
mOMTN
mSert
mGaba2
mGaba1a
mGaba1b
mRN
mNbML4
mNbL1
mNbML3
mNbML1
mDA2
mDA1
mDA0
mNbDA
mNbL2
mNbML2
mNbM
mNProg
mRgl1
mRgl2
mRgl3
mEpend
mMgl
mPeric
mEndo
Correlation Coefficient
0.0
0.5

**Supplementary Figure 1. Comparison of mouse and human midbrain using HomologySeeker**

**a)** Schematic representation of the selection process for Homologous-HVGs. **b)** Selection of Homologous-HVGs from *mouse* and *human* midbrain datasets sourced from (Manno et al., 2016). Red dots represent homologous genes that have a standardized variance above the mean value for all genes. **c)** Intersection of Homologous-HVGs sets between *mouse* and *human* as derived in (b). **d)** Overlap between Homologous-HVGs and the set of 1405 genes from La Manno et al. **e)** Correlation of Cell types between *mouse* and *human* datasets from La Manno et al using Homologous-HVGs. Rows represent *mouse* cell types, while columns represent *human* cell types.

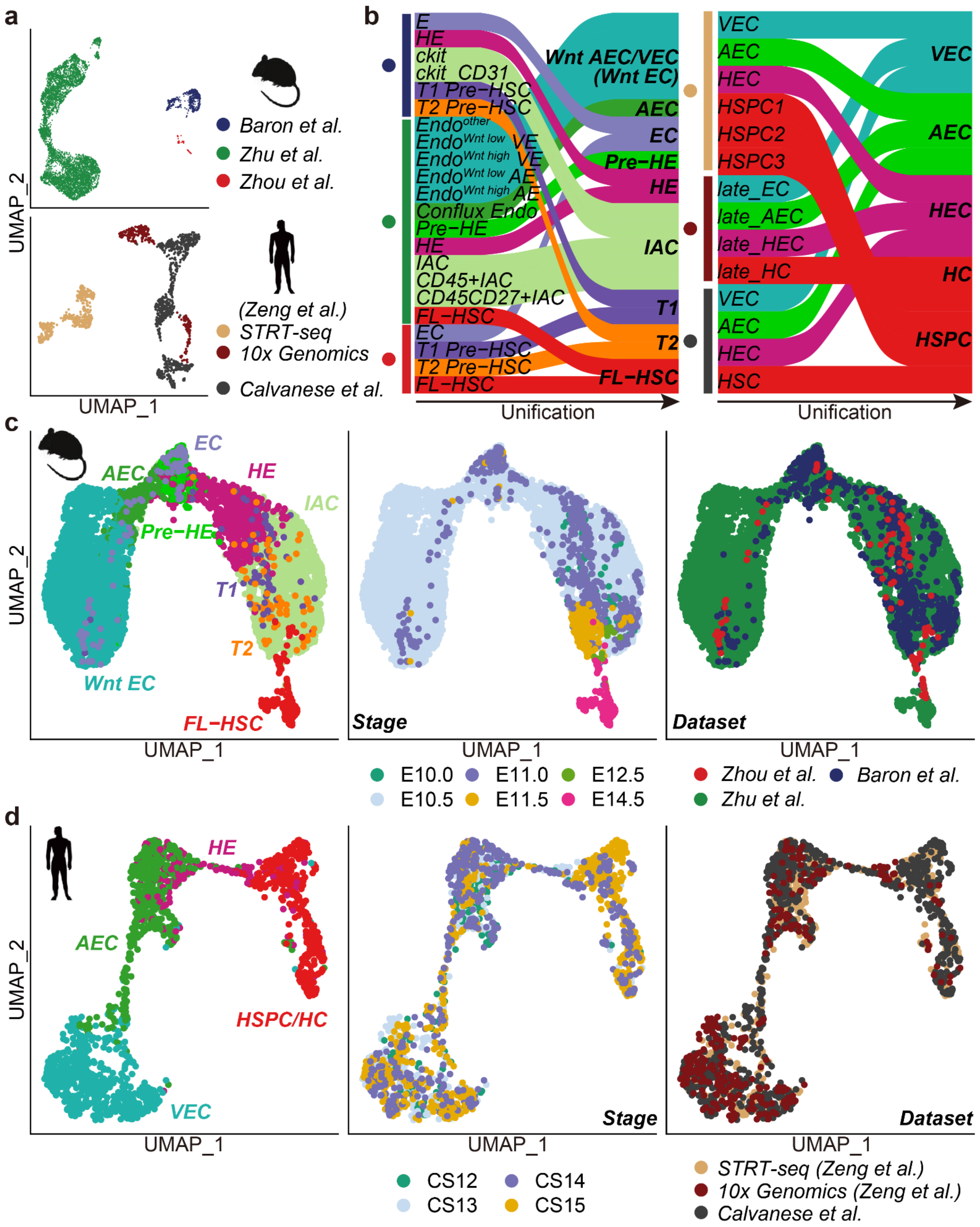
a
UMAP_2
UMAP_1
Baron et al.
Zhu et al.
Zhou et al.
(Zeng et al.)
STRT-seq
10x Genomics
Calvanese et al.
b
E
HE
ckit
ckit_CD31
T1 Pre-HSC
T2 Pre-HSC
Endo^other
Endo^Wnt low VE
Endo^Wnt high VE
Endo^Wnt low AE
Endo^Wnt high AE
Conflux Endo
Pre-HE
HE
IAC
CD45+IAC
CD45CD27+IAC
FL-HSC
EC
T1 Pre-HSC
T2 Pre-HSC
FL-HSC
Wnt AEC/VEC (Wnt EC)
AEC
EC
Pre-HE
HE
IAC
T1
T2
FL-HSC
Unification
VEC
AEC
HEC
HSPC1
HSPC2
HSPC3
late_EC
late_AEC
late_HEC
late_HC
VEC
AEC
HEC
HSC
VEC
AEC
HEC
HC
HSPC
Unification
c
EC
AEC
HE
IAC
Pre-HE
T1
T2
Wnt EC
FL-HSC
Stage
Dataset
UMAP_2
UMAP_1
E10.0
E11.0
E12.5
E10.5
E11.5
E14.5
Zhou et al.
Baron et al.
Zhu et al.
d
HE
AEC
HSPC/HC
VEC
Stage
Dataset
UMAP_2
UMAP_1
CS12
CS14
CS13
CS15
STRT-seq (Zeng et al.)
10x Genomics (Zeng et al.)
Calvanese et al.

**Supplementary Figure 2. Construction of EHT ensembles**

**a)** UMAP representations of *mouse* (upper panel) and *human* (lower panel) scRNA-seq data prior to integration. Each dot corresponds to a single cell, and colors indicate different datasets. **b)** Unification of cell identity. The left panel displays the *mouse* annotation, while the right panel shows the *human* annotation. **c-d)** The constructed EHT ensembles for *mouse* (c) and *human* (d). Colors denote unified identities (left panel), cell stages (middle panel), and originating datasets (right panel).

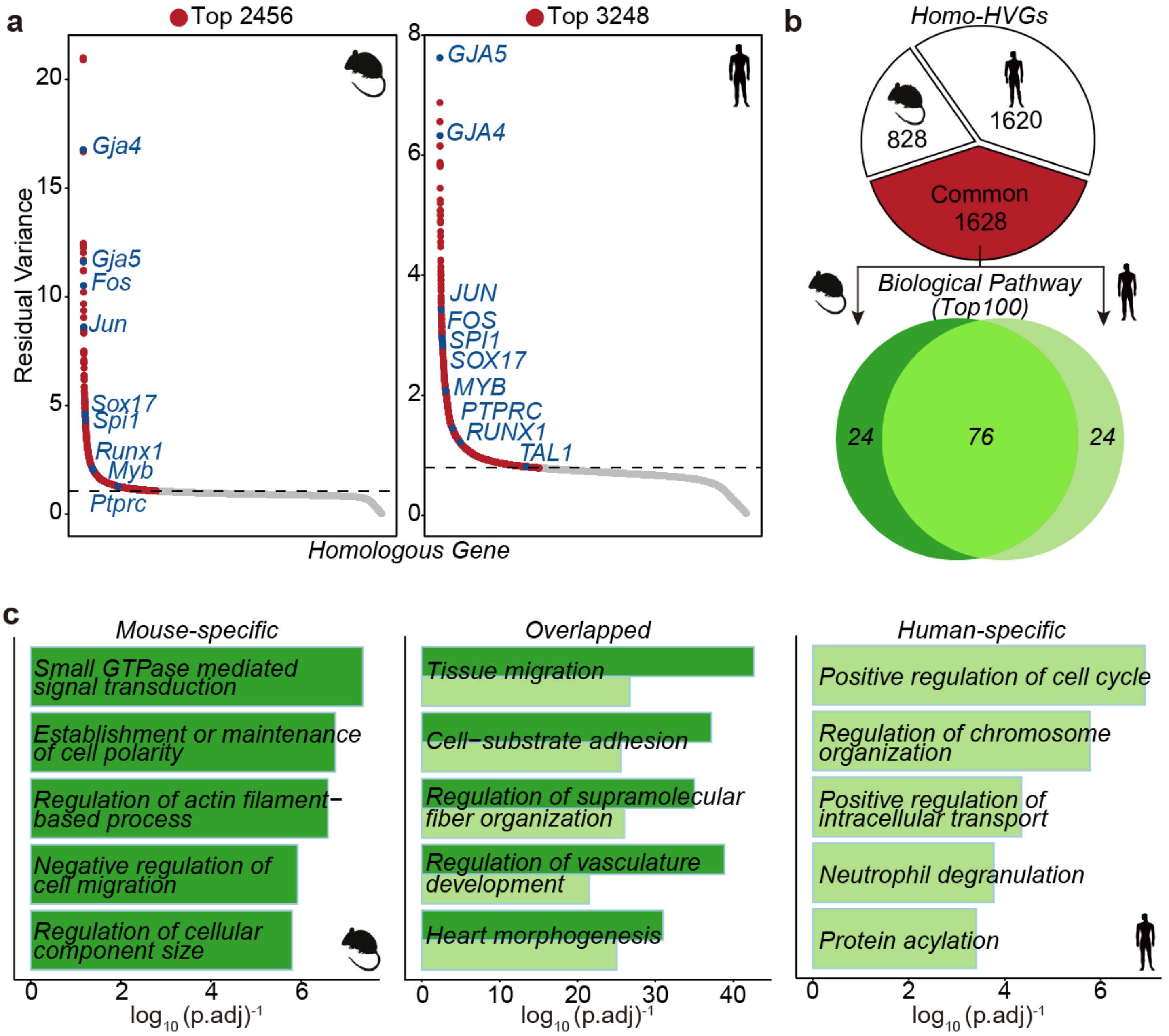


**Supplementary Figure 3. Identification of Homologous-HVGs in EHT ensembles**

**a)** Selection of Homologous-HVGs in *mouse* and *human* EHT ensembles. Red dots represent homologous genes above the cut off, while blue dots signify EHT-related genes. The dashed line indicates the mean value of the residual variance for all homologous genes. **b)** Intersection of Homologous-HVGs sets and enriched biological pathways. The upper panel depicts the overlap between *mouse* and *human* Homologous-HVGs sets, while the lower panel shows the overlap among the top 100 biological pathways enriched by *mouse* and *human* Homologous-HVGs. **c)** The top 5 biological pathways derived from different sections of the Venn diagram in (b).

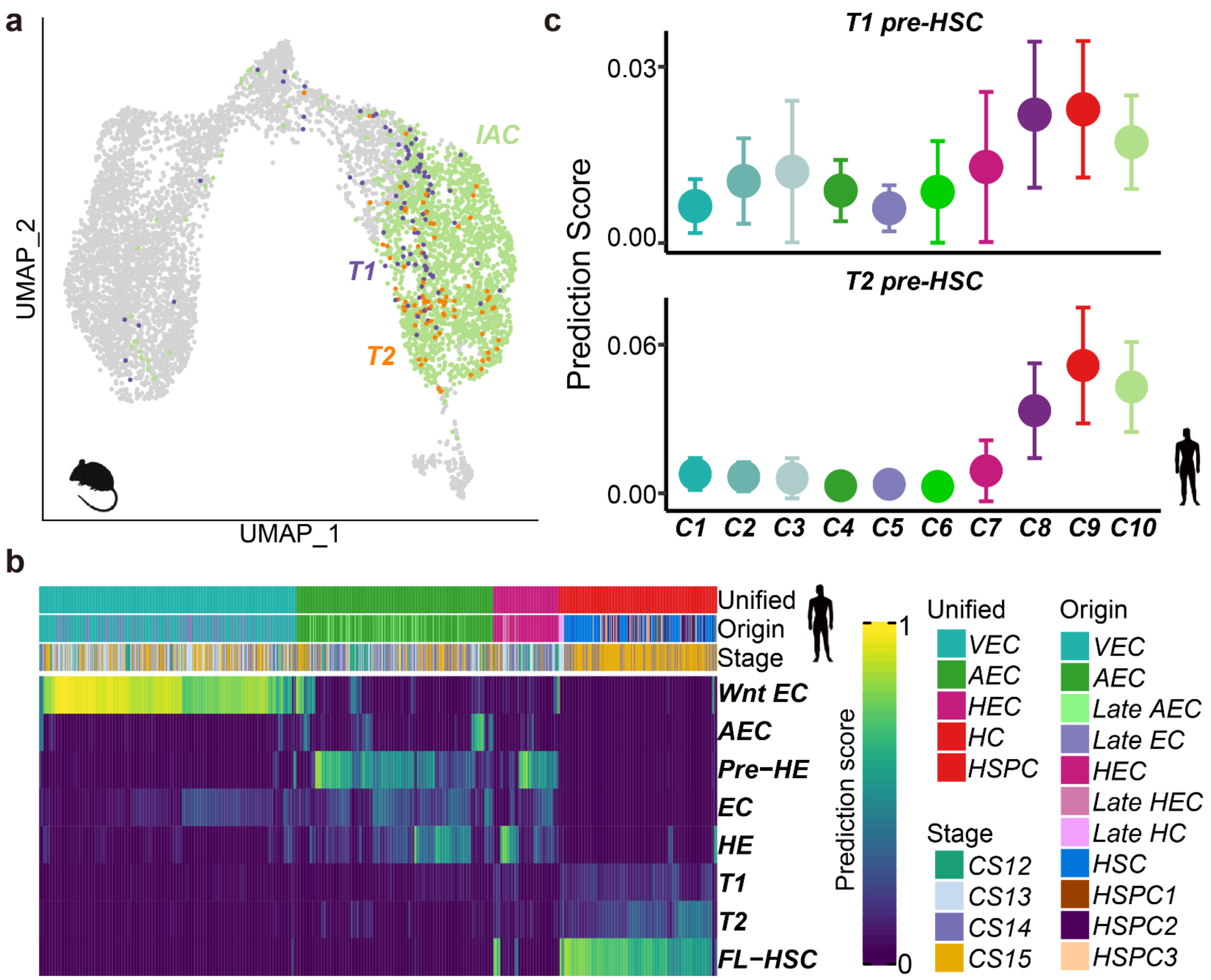


**Supplementary Figure 4. Query projection without mouse IAC in the reference**

**a)** Depiction of T1, T2, and IAC positions within the *mouse* EHT ensemble. T1 and T2 represent pre-HSC, while IAC stands for intra-aortic cluster cells. **b)** Heatmap illustrating the projection scores of *human* cell types assigned with new identities from *mouse* through query projection, excluding IAC cells. Each column represents a cell from the *human* ensemble, each row corresponds to a *mouse* cell type, and the heatmap displays the prediction score of each *human* cell using the *mouse* as a reference. "Unified" refers to the unified *human* cell type annotation. "Origin" denotes the origin cell type annotation from *human* datasets. "Stage" indicates the timepoints of each cell. **c)** SingleCellNet prediction scores of *human* cells with a re-scaled y-axis from Figure 1f. A higher score indicates greater prediction confidence.

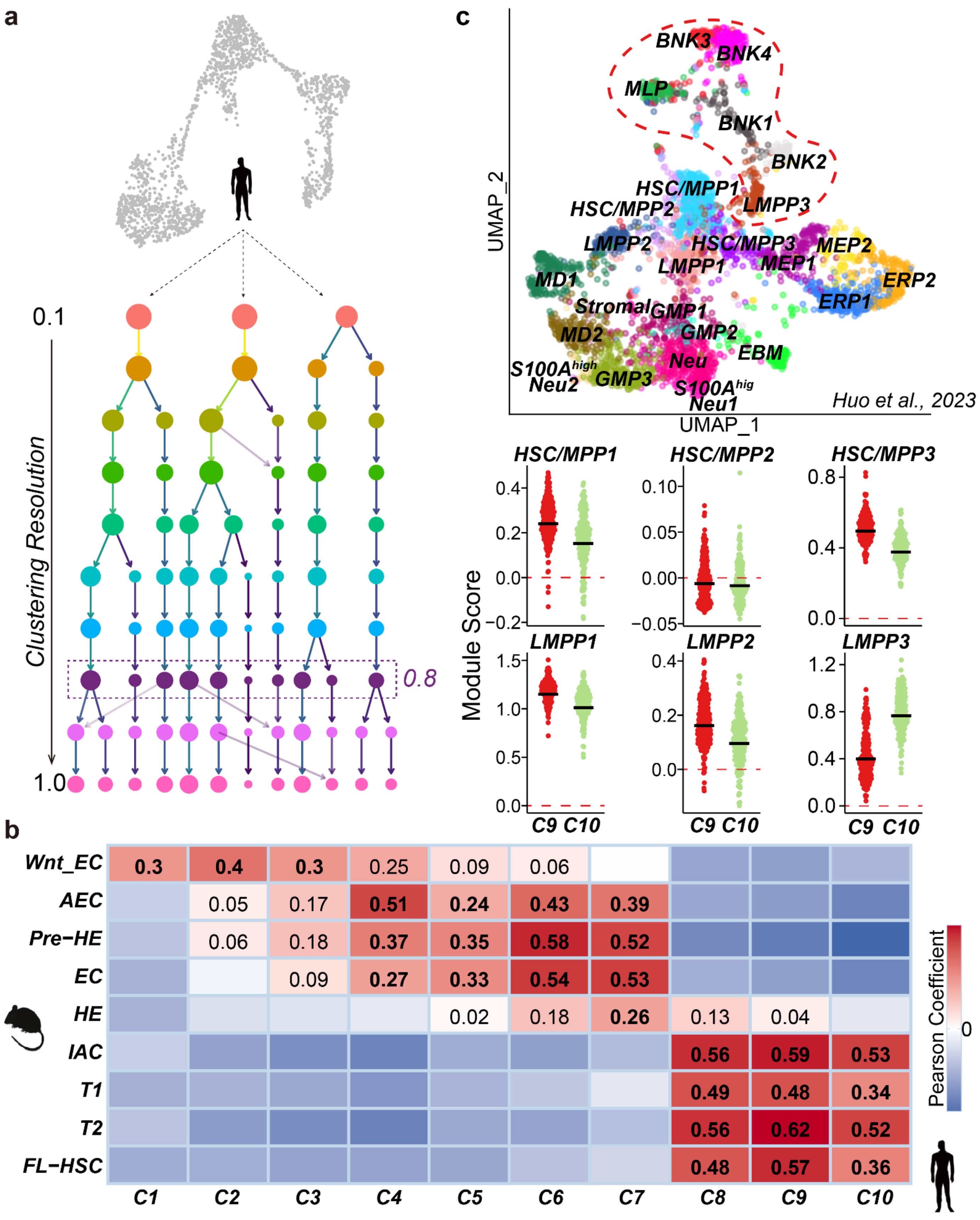
a
0.1
Clustering Resolution
0.8
1.0
c
BNK3
BNK4
MLP
BNK1
BNK2
HSC/MPP1
HSC/MPP2
LMPP3
LMPP2
HSC/MPP3
MEP2
LMPP1
MEP1
MD1
ERP2
ERP1
Stromal
GMP1
MD2
GMP2
EBM
Neu
S100A^high Neu2
GMP3
S100A^hig Neu1
UMAP_2
UMAP_1
Huo et al., 2023
HSC/MPP1
HSC/MPP2
HSC/MPP3
LMPP1
LMPP2
LMPP3
Module Score
C9 C10
C9 C10
C9 C10
b
Wnt_EC
AEC
Pre−HE
EC
HE
IAC
T1
T2
FL−HSC
0.3 0.4 0.3 0.25 0.09 0.06
0.05 0.17 0.51 0.24 0.43 0.39
0.06 0.18 0.37 0.35 0.58 0.52
0.09 0.27 0.33 0.54 0.53
0.02 0.18 0.26 0.13 0.04
0.56 0.59 0.53
0.49 0.48 0.34
0.56 0.62 0.52
0.48 0.57 0.36
C1 C2 C3 C4 C5 C6 C7 C8 C9 C10
Pearson Coefficient
0

**Supplementary Figure 5. Unsupervised clustering of human EHT ensembles**

**a)** Cluster tree representing clustering resolution. Each circle represents to an individual subcluster, and the number of the circles with the same color indicates the resulting number of subclusters under a specific clustering resolution. Arrows indicate the reassignment of cells among subclusters. **b)** Pearson correlation between *mouse* cell types and *human* subclusters. Colors represent Pearson correlation coefficients, with only positive coefficients shown. **c)** Upper UMAP: UMAP of hematopoietic stem/progenitors cells derived from bone marrow or peripheral blood, as reported in Huo et al., 2023. The red circle encompasses the developmental trajectory starting from LMPP3. Lower panel: Module score relating to different HSC/MPP and LMPP clusters.

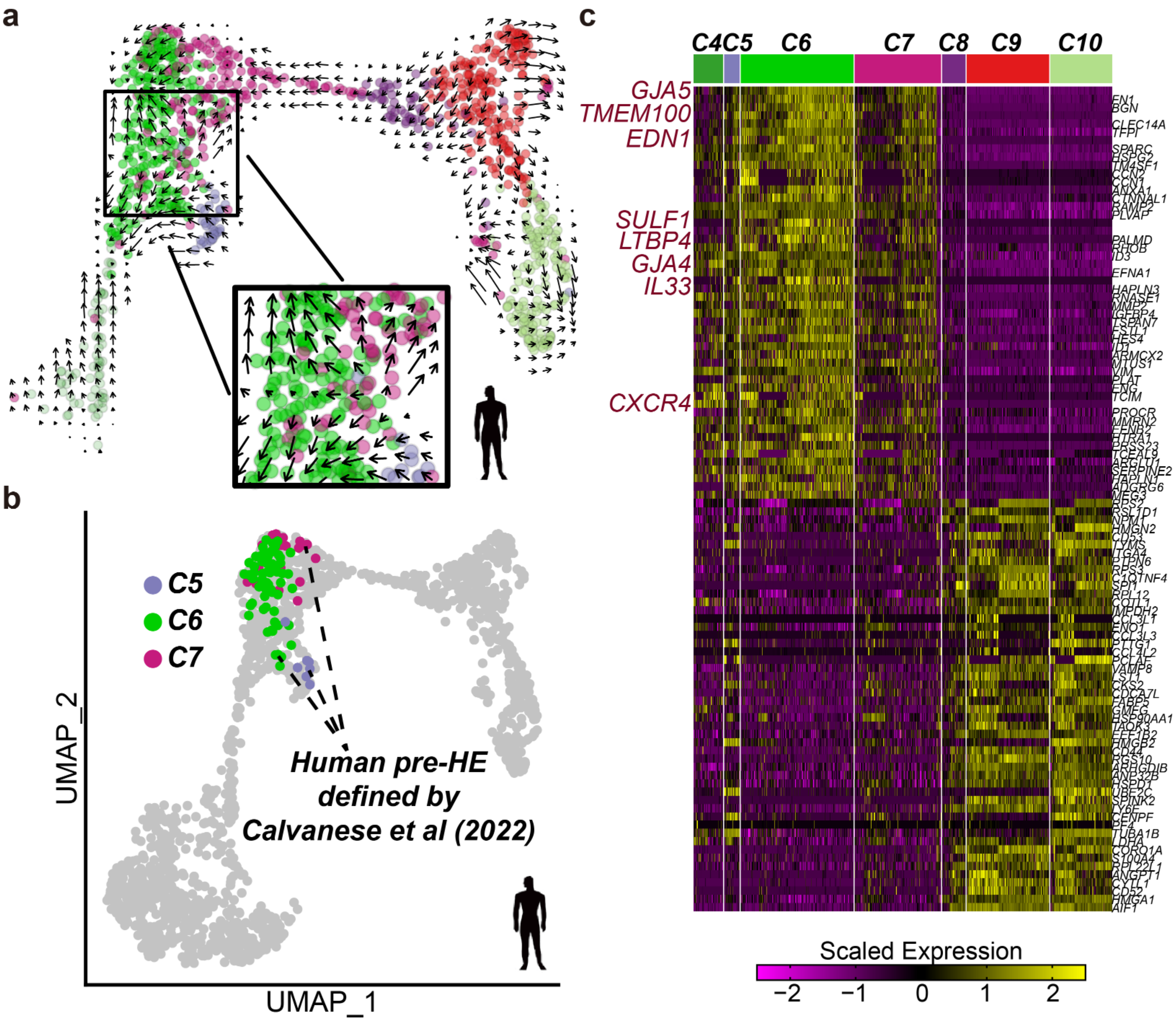


**Supplementary Figure 6. Human C6 subcluster is characterized by pre-HE signatures**

**a)** RNA velocity of the *human* EHT ensemble (excluding the venous EC group: C1-C3). Arrows indicate different differentiation dynamics among *human* cells. **b)** Single cells classified as pre-HEs by Calvanese et al. Since the *human* EHT ensembles incorporate the single cell dataset from Calvanese et al., highlighted cells are colored according to *human* subclusters. **c)** Heatmap showing the scaled expression of DEGs in *human* C6 compared to all other subclusters. Only the top 50 up- and down-regulated DEGs are shown.

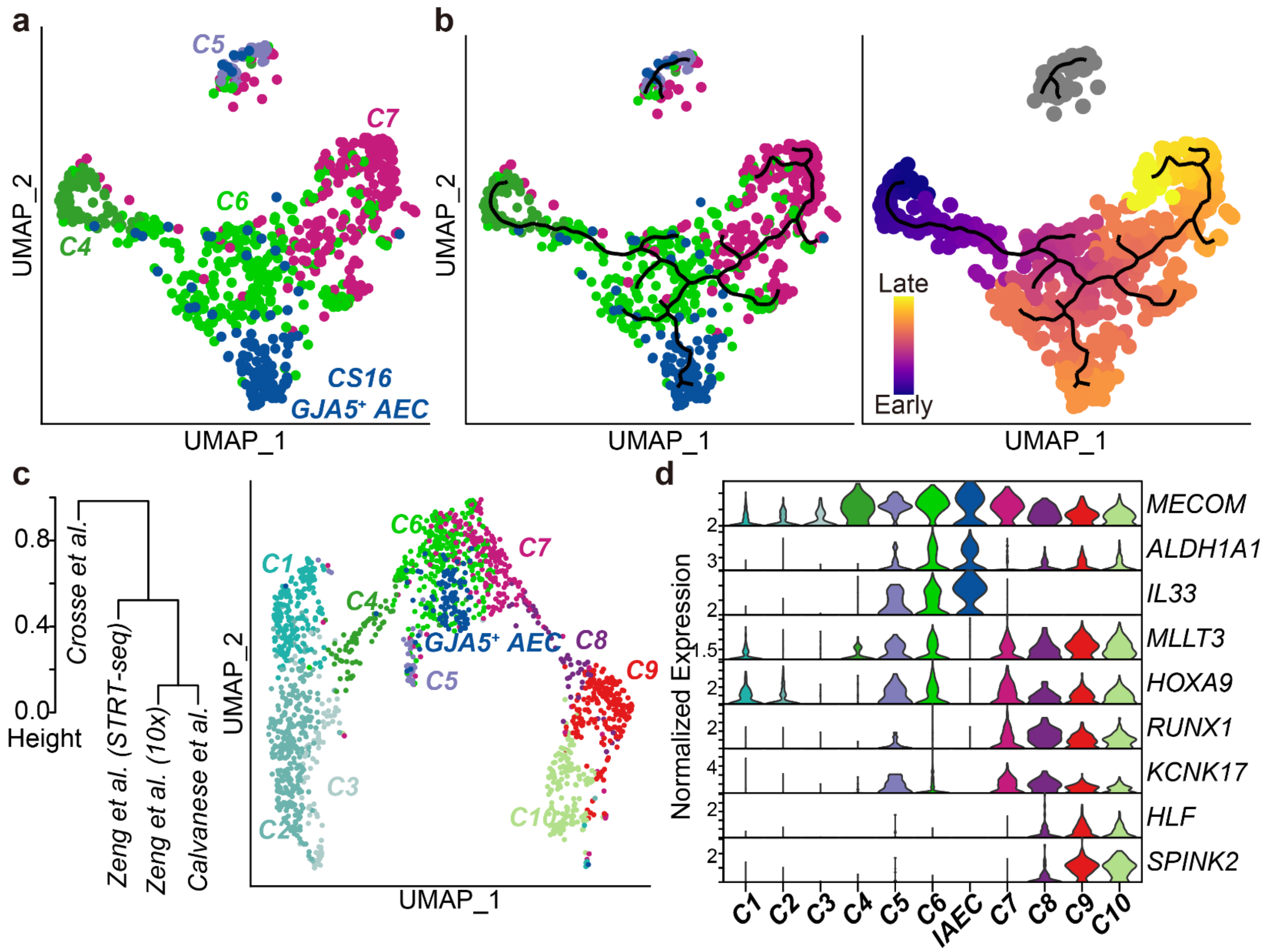


**Supplementary Figure 7. Integration of GJA5⁺ AECs into the human EHT ensemble**

**a)** UMAP visualization of the integration of GJA5⁺ AECs with *human* C4-C7. **b)** Monocle3 trajectory analysis of the combined landscape of GJA5⁺ AECs and *human* C4-C7. Left UMAP depicts the developmental trajectory of the combined landscape, while the right UMAP illustrates the pseudo-time progression along the developmental trajectory. **c)** Left panel displays an integration tree used as pairwise similarity criteria for samples integration. Right UMAP shows the integration of GJA5⁺ AECs into the *human* EHT ensemble. **d)** Expression of EHT key genes from Calvanese et al. within lAEC and GJA5⁺ AEC populations.

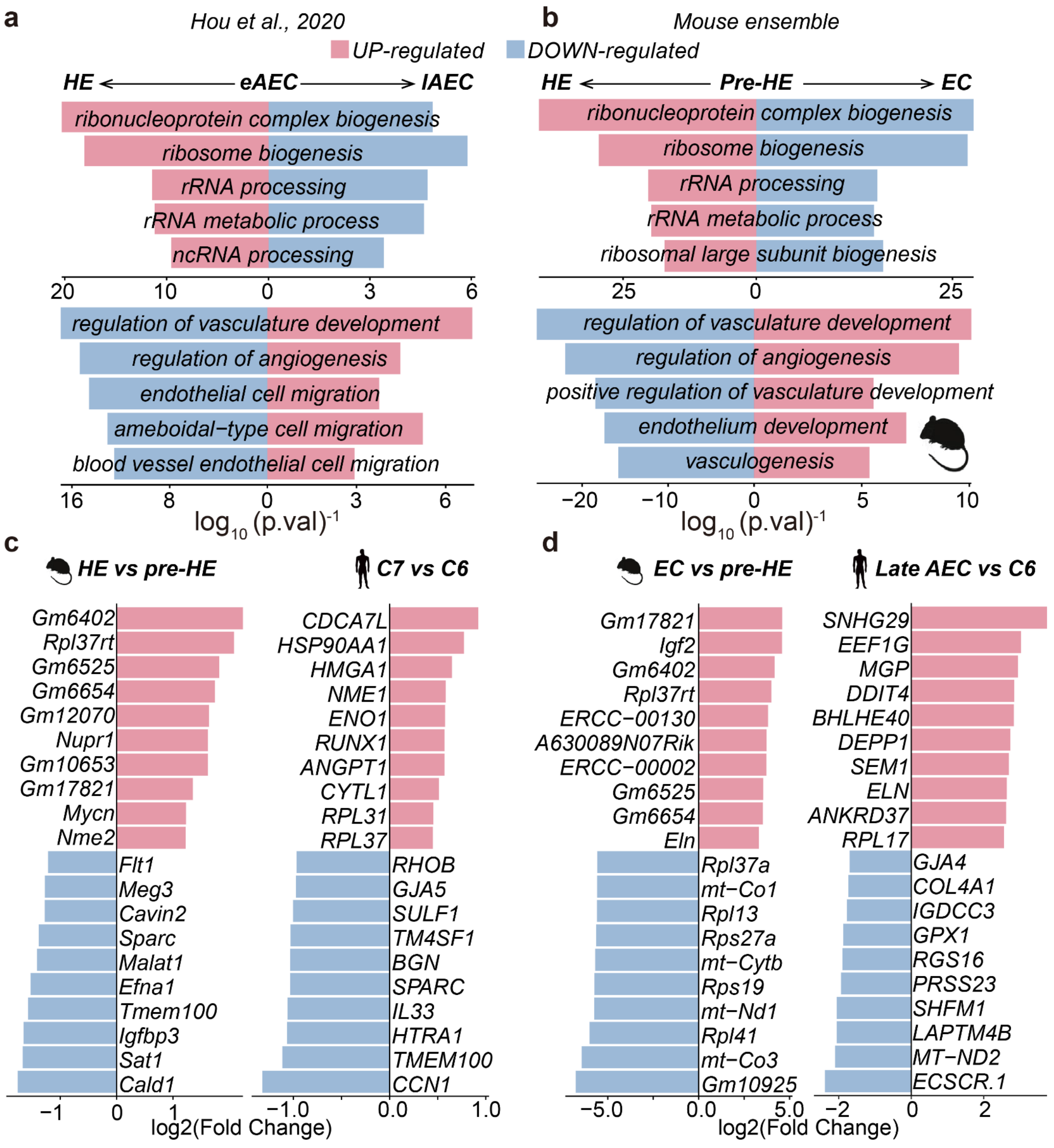

a
Hou et al., 2020
UP-regulated
DOWN-regulated
HE
eAEC
lAEC
ribonucleoprotein complex biogenesis
ribosome biogenesis
rRNA processing
rRNA metabolic process
ncRNA processing
regulation of vasculature development
regulation of angiogenesis
endothelial cell migration
ameboidal−type cell migration
blood vessel endothelial cell migration
log10 (p.val)-1
b
Mouse ensemble
HE
Pre-HE
EC
ribonucleoprotein complex biogenesis
ribosome biogenesis
rRNA processing
rRNA metabolic process
ribosomal large subunit biogenesis
regulation of vasculature development
regulation of angiogenesis
positive regulation of vasculature development
endothelium development
vasculogenesis
log10 (p.val)-1
c
HE vs pre-HE
C7 vs C6
Gm6402
Rpl37rt
Gm6525
Gm6654
Gm12070
Nupr1
Gm10653
Gm17821
Mycn
Nme2
Flt1
Meg3
Cavin2
Sparc
Malat1
Efna1
Tmem100
Igfbp3
Sat1
Cald1
CDCA7L
HSP90AA1
HMGA1
NME1
ENO1
RUNX1
ANGPT1
CYTL1
RPL31
RPL37
RHOB
GJA5
SULF1
TM4SF1
BGN
SPARC
IL33
HTRA1
TMEM100
CCN1
log2(Fold Change)
d
EC vs pre-HE
Late AEC vs C6
Gm17821
Igf2
Gm6402
Rpl37rt
ERCC−00130
A630089N07Rik
ERCC−00002
Gm6525
Gm6654
Eln
Rpl37a
mt−Co1
Rpl13
Rps27a
mt−Cytb
Rps19
mt−Nd1
Rpl41
mt−Co3
Gm10925
SNHG29
EEF1G
MGP
DDIT4
BHLHE40
DEPP1
SEM1
ELN
ANKRD37
RPL17
GJA4
COL4A1
IGDCC3
GPX1
RGS16
PRSS23
SHFM1
LAPTM4B
MT−ND2
ECSCR.1
log2(Fold Change)

**Supplementary Figure 8. Differential expression analysis between pre-HE/early AEC and HE/late AEC**

**a)** The top 5 biological pathways enriched by differentially expressed genes (DEGs) between *mouse* early AEC and HE, alongside the identical terms enriched by DEGs between early AEC and Late AEC from Hou et al. GO terms in red and blue represent enrichment by up- and down-regulated DEGs, respectively. **b)** The top 5 biological pathways enriched by DEGs between *mouse* pre-HE and HE, along with identical terms enriched by DEGs between pre-HE and EC from EHT ensemble. GO terms in red and blue denote enrichment by up- and down-regulated DEGs, respectively. **c)** On the left, the top 10 DEGs between *mouse* HE and pre-HE are shown, while on the right, the top 10 DEGs between *human* C7 and C6 are displayed. DEGs are ordered according to fold change. **d)** On the left, the top 10 DEGs between *mouse* EC and pre-HE are presented, and on the right, the top 10 DEGs between *human* late AEC ($GJA5^+$ AEC) and C6. DEGs are ordered by fold change.

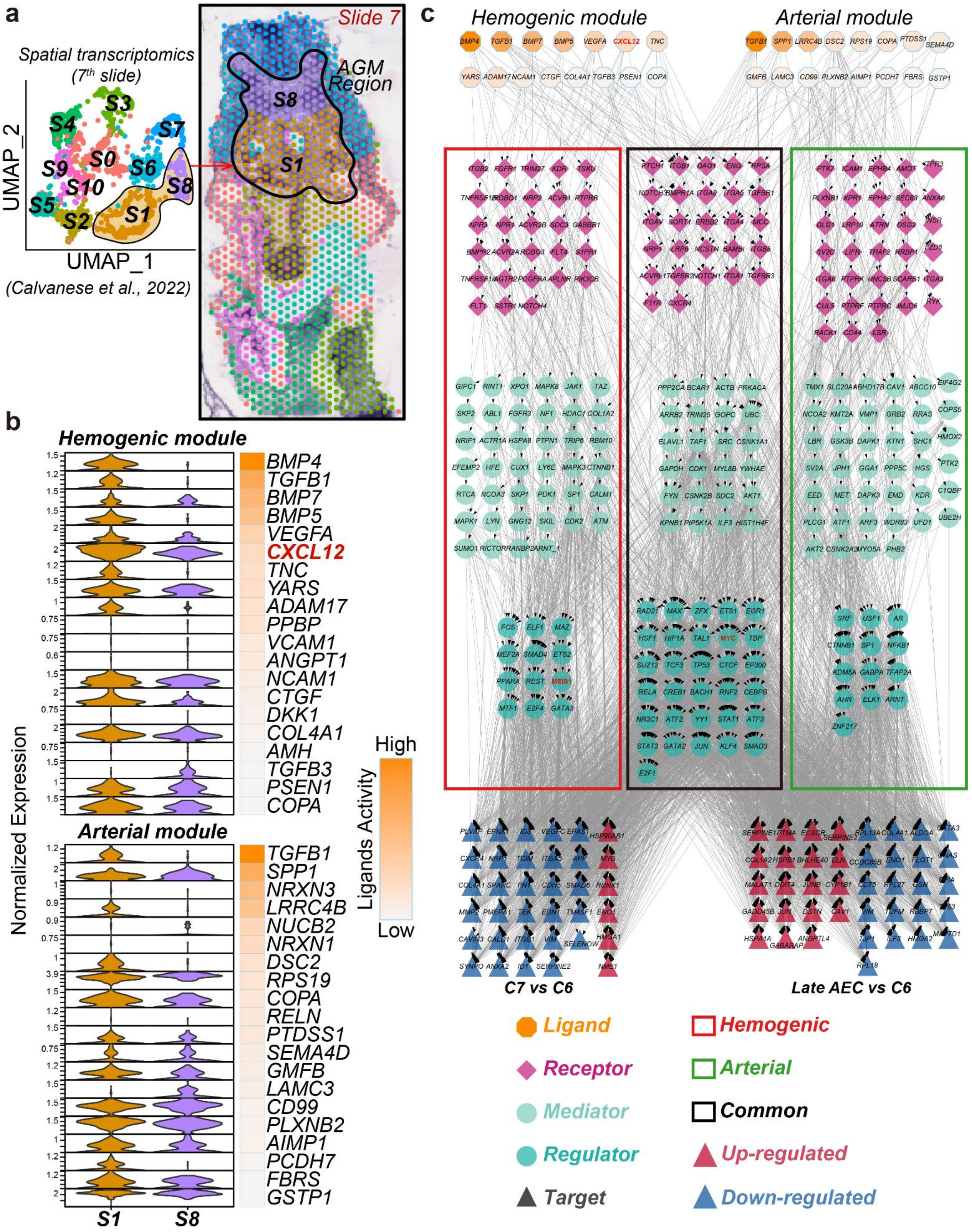
a
Spatial transcriptomics (7th slide)
Slide 7
AGM Region
S0
S1
S2
S3
S4
S5
S6
S7
S8
S9
S10
UMAP_1
UMAP_2
(Calvanese et al., 2022)
b
Hemogenic module
BMP4
TGFB1
BMP7
BMP5
VEGFA
CXCL12
TNC
YARS
ADAM17
PPBP
VCAM1
ANGPT1
NCAM1
CTGF
DKK1
COL4A1
AMH
TGFB3
PSEN1
COPA
Arterial module
TGFB1
SPP1
NRXN3
LRRC4B
NUCB2
NRXN1
DSC2
RPS19
COPA
RELN
PTDSS1
SEMA4D
GMFB
LAMC3
CD99
PLXNB2
AIMP1
PCDH7
FBRS
GSTP1
Normalized Expression
Ligands Activity
High
Low
S1
S8
c
Hemogenic module
Arterial module
C7 vs C6
Late AEC vs C6
Ligand
Receptor
Mediator
Regulator
Target
Hemogenic
Arterial
Common
Up-regulated
Down-regulated

**Supplementary Figure 9. Identification of signaling pathways mediated by ligand modules**

**a)** Unsupervised clustering of spatial transcriptomics data sequenced from CS15 *human* embryo slide (Calvanese et al., 2022, 7th Slide). Each dot in the UMAP represents a single spot, colors corresponding to different clusters. **b)** Expression of top 20 potential ligands that shared active links with target genes (DEGs). Ligand activity is shown in orange. Hemogenic/arterial module, ligands module that facilitate hemogenic or arterial choice of pre-HE, respectively. **c)** Signaling pathways mediated by potential ligand modules that may facilitate cell fate choice of *human* C6. Pathways cover the path's start from ligands (orange) to receptors (pink), through signaling mediators (light blue) and regulators (blue), finally ending at targets (DEGs). Red, black, and green rectangles represent specific (red and green) or shared (black) components involved in signaling pathways. Target genes in burgundy refer to up-regulated genes. Target genes in blue refer to down-regulated genes.

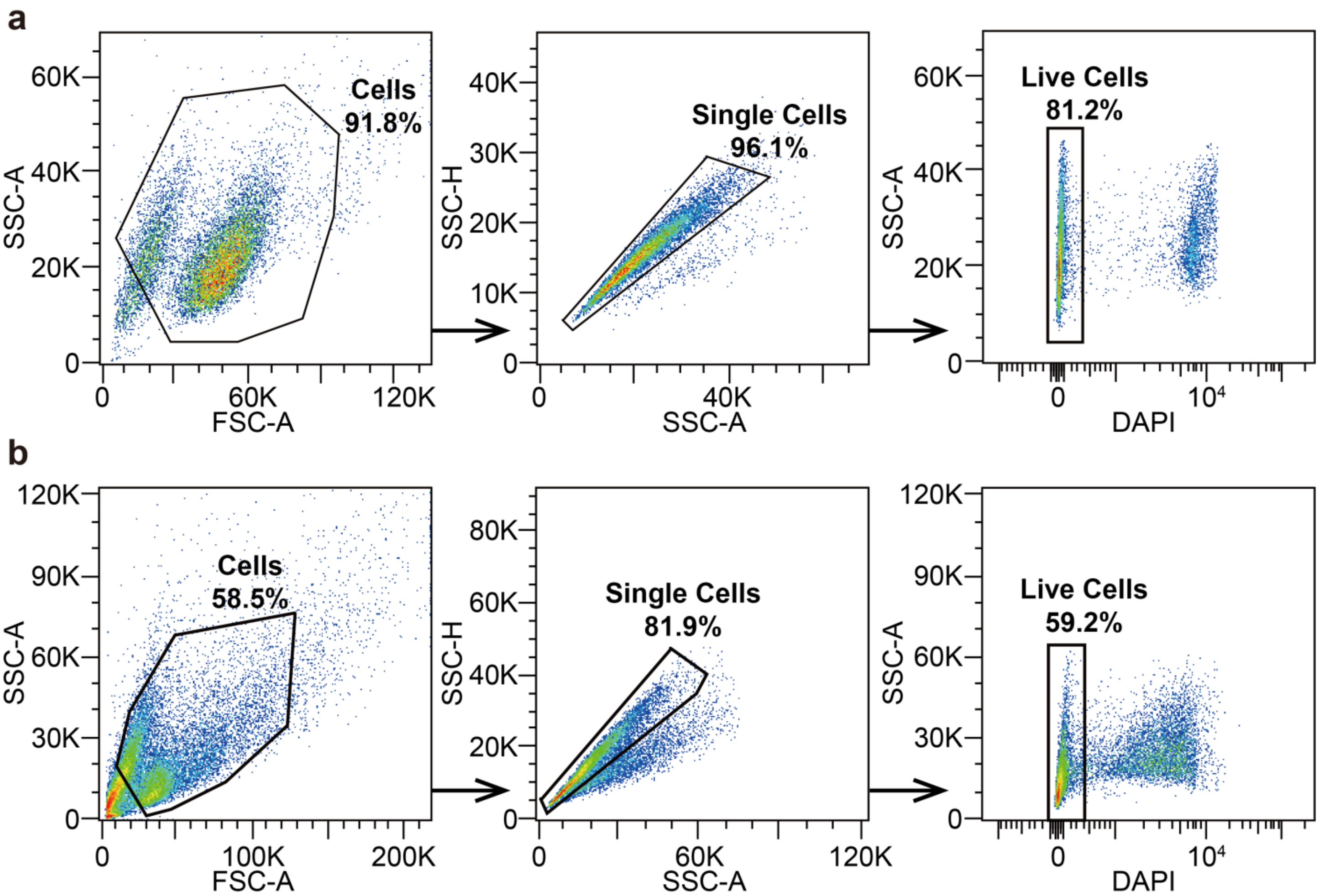


**Supplementary Figure 10. Gating strategy for flow cytometry analysis of *in vitro* defined HE and HPC**

gating strategy for flow cytometry analysis of in vitro defined HE (a) and HPC (b). HE, hemogenic endothelium; HPC, hematopoietic progenitor cell.
