# Supplementary material for: Cross-species transcriptomics reveals bifurcation point during the arterial-to-hemogenic transition
Source: Commun Biol. 2023 Aug 9;6:827. doi: 10.1038/s42003-023-05190-6 (PMC10412572; doi:10.1038/s42003-023-05190-6)
Supplement: Supplementary file 3 — Description of Additional Supplementary Files [file 42003_2023_5190_MOESM3_ESM.pdf]

Description of Additional Supplementary Files

File name: Supplementary Data

Description: The source data contains gene sets, biological terms, singling pathways components, primer sequences, and cell metadata mentioned in the paper.
