# Supplementary material for: Cross-species transcriptomics reveals bifurcation point during the arterial-to-hemogenic transition
Source: Commun Biol. 2023 Aug 9;6:827. doi: 10.1038/s42003-023-05190-6 (PMC10412572; doi:10.1038/s42003-023-05190-6)
Supplement: Supplementary file 5 — Reporting Summary [file 42003_2023_5190_MOESM5_ESM.pdf]

nature portfolio

Corresponding author(s): Kuangyu Yen

Last updated by author(s): Jul 19, 2023

Reporting Summary

Nature Portfolio wishes to improve the reproducibility of the work that we publish. This form provides structure for consistency and transparency in reporting. For further information on Nature Portfolio policies, see our Editorial Policies and the Editorial Policy Checklist.

Statistics

For all statistical analyses, confirm that the following items are present in the figure legend, table legend, main text, or Methods section.

n/a	Confirmed	
☐	☒	The exact sample size (*n*) for each experimental group/condition, given as a discrete number and unit of measurement
☐	☒	A statement on whether measurements were taken from distinct samples or whether the same sample was measured repeatedly
☐	☒	The statistical test(s) used AND whether they are one- or two-sided<br>*Only common tests should be described solely by name; describe more complex techniques in the Methods section.*
☒	☐	A description of all covariates tested
☒	☐	A description of any assumptions or corrections, such as tests of normality and adjustment for multiple comparisons
☐	☒	A full description of the statistical parameters including central tendency (e.g. means) or other basic estimates (e.g. regression coefficient) AND variation (e.g. standard deviation) or associated estimates of uncertainty (e.g. confidence intervals)
☐	☒	For null hypothesis testing, the test statistic (e.g. *F*, *t*, *r*) with confidence intervals, effect sizes, degrees of freedom and *P* value noted<br>*Give P values as exact values whenever suitable.*
☒	☐	For Bayesian analysis, information on the choice of priors and Markov chain Monte Carlo settings
☒	☐	For hierarchical and complex designs, identification of the appropriate level for tests and full reporting of outcomes
☒	☐	Estimates of effect sizes (e.g. Cohen's *d*, Pearson's *r*), indicating how they were calculated

Our web collection on statistics for biologists contains articles on many of the points above.

Software and code

Policy information about availability of computer code

Data collection	All available single-cell RNA sequencing datasets, spatial transcriptomics dataset and meta data used in this study were collected from Gene Expression Omnibus (GEO) repository using Aspera (v3.9.8.176272) and from GitHub repository. Genome sequences of human and mouse were collected from Ensembl (https://www.ensembl.org/index.html). Flow cytometry data was collected using Canto II analyzer (BD Biosciences). Data from quantitative real-time polymerase chain reaction (qRT-PCR) were obtained using QuantStudio™ 3 (Applied Biosystems).
Data analysis	10X Genomics software cellranger (v2.1.1), trim_galore (v0.6.7), STAR (v2.6.0c), HTSeq (v0.13.5), R packages under R environment (v4.0.3): limma (v3.18.10), biomaRt (v2.46.3), Seurat (v4.1.1), sctransform(v0.3.4), STACAS (v2.0.1), clustree (v0.4.4), clusterProfiler (v4.5.0.992), Monocle (v2.9.0), Monocle3 (v1.2.7), ggplot2 (v3.3.6), dynamicTreeCut (v1.63-1), SingleCellNet (v0.1.0), Velocyto (v0.17.17), velocyto.R (v0.6), NicheNet (v1.1.0), OmnipathR (v3.5.21), Cytoscape (v3.8.2) and ComplexHeatmap (v2.6.2)were used to analyze the single-cell RNA sequencing and spatial transcriptomics datasets. Source code for HomologySeeker are deposited in GitHub (https://github.com/YenLab/HomologySeeker). Interfaces for mouse and human EHT ensembles are deposited using VisCello (v1.1.2). Flow cytometry analysis was performed using FlowJo software (V10).

For manuscripts utilizing custom algorithms or software that are central to the research but not yet described in published literature, software must be made available to editors and reviewers. We strongly encourage code deposition in a community repository (e.g. GitHub). See the Nature Portfolio guidelines for submitting code & software for further information.

Data

Policy information about availability of data

All manuscripts must include a data availability statement. This statement should provide the following information, where applicable:

- Accession codes, unique identifiers, or web links for publicly available datasets
- A description of any restrictions on data availability
- For clinical datasets or third party data, please ensure that the statement adheres to our policy

All single-cell RNA sequencing datasets, spatial transcriptomics dataset used in this study are publicly available in Gene Expression Omnibus (GEO) repository under accession numbers: (Zhou et al., 2016, GSE135202), (Baron et al., 2018, GSE112642), (Zhu et al., 2020, GSE137117), (Hou et al., 2020, GSE139389), (Zeng et al., 2019, GSE135202), (Crosse et al., 2020, GSE151877), (Calvanese et al., 2022, GSE162950), (La Manno et al., 2016, GSE76381), (Huo et al., 2023, GSE224714). Interfaces for mouse (https://soap4.shinyapps.io/mouse_ensemble/?_ga=2.41033381.689702864.1670560928-554826141.1655946746) and human (https://soap4.shinyapps.io/human_ensemble/?_ga=2.99744409.689702864.1670560928-554826141.1655946746) EHT ensembles are deposited at shinyapps.io. There is no restriction in data availability.

Research involving human participants, their data, or biological material

Policy information about studies with human participants or human data. See also policy information about sex, gender (identity/presentation), and sexual orientation and race, ethnicity and racism.

Reporting on sex and gender	No sex- or gender-based analysis was performed in this study.
Reporting on race, ethnicity, or other socially relevant groupings	No race, ethnicity, or other socially relevant groupings were involved in this study.
Population characteristics	See above
Recruitment	No participants were involved in this study.
Ethics oversight	This study does not address ethics restrictions.

Note that full information on the approval of the study protocol must also be provided in the manuscript.

Field-specific reporting

Please select the one below that is the best fit for your research. If you are not sure, read the appropriate sections before making your selection.

☒ Life sciences ☐ Behavioural & social sciences ☐ Ecological, evolutionary & environmental sciences

For a reference copy of the document with all sections, see nature.com/documents/nr-reporting-summary-flat.pdf

Life sciences study design

All studies must disclose on these points even when the disclosure is negative.

Sample size	The sample size was chosen based on previous publications. No statistical test was performed to predetermine the sample size.
Data exclusions	For each dataset, only cells or spots with pre-defined annotations were obtained based on previous publications.
Replication	Each experiment was performed with multiple biological and technical replicates at least thrice.
Randomization	Cells were randomly chosen from the human H1 pluripotent stem cell population for each experiment.
Blinding	Blinding was not applicable, as all datasets used in this study were publicly available.

Reporting for specific materials, systems and methods

We require information from authors about some types of materials, experimental systems and methods used in many studies. Here, indicate whether each material, system or method listed is relevant to your study. If you are not sure if a list item applies to your research, read the appropriate section before selecting a response.

Materials & experimental systems

n/a	Involved in the study	
☐	☒	Antibodies
☐	☒	Eukaryotic cell lines
☒	☐	Palaeontology and archaeology
☒	☐	Animals and other organisms
☒	☐	Clinical data
☒	☐	Dual use research of concern
☒	☐	Plants

Methods

n/a	Involved in the study	
☒	☐	ChIP-seq
☐	☒	Flow cytometry
☒	☐	MRI-based neuroimaging

Antibodies

Antibodies used

CD73-PE-Cy7 (BioLegend, clone: AD2)
CD184-APC (Invitrogen, clone: 12G5)
CD144-PE (Invitrogen, clone: 16B1)
CD34-APC-Cy7 (BioLegend, clone: 561)
CD34-PE (Invitrogen, clone: 4H11)
CD43-PE (BioLegend, clone: 10G7)

Validation

All antibodies for flow cytometry are commercially available and were validated based on information provided by the manufacturer.

Eukaryotic cell lines

Policy information about cell lines and Sex and Gender in Research

Cell line source(s)

The H1 pluripotent stem cell line was obtained from the WiCell Research Institute (Madison, WI, http://www.wicell.org).

Authentication

The H1 pluripotent stem cell line used in this study was authenticated by the WiCell Research Institute.

Mycoplasma contamination

All cell lines were tested negative for mycoplasma infection.

Commonly misidentified lines
(See ICLAC register)

No commonly misidentified lines were used.

Flow Cytometry

Plots

Confirm that:

☒ The axis labels state the marker and fluorochrome used (e.g. CD4-FITC).

☒ The axis scales are clearly visible. Include numbers along axes only for bottom left plot of group (a 'group' is an analysis of identical markers).

☒ All plots are contour plots with outliers or pseudocolor plots.

☒ A numerical value for number of cells or percentage (with statistics) is provided.

Methodology

Sample preparation

Cultured cells were maintained on Matrigel-coated 6-well plates (Corning) containing E8 medium (Gibco) and the medium was replaced daily. The hPSCs were sub-cultured every 3-4 days with a treatment of 0.5 mM ethylenediaminetetraacetic acid (EDTA; Life Technologies) for passaging when cells reached 60% to 70% confluence. single-cell suspensions of hPSCs were obtained by treating the hPSC cultures at 70%-80% confluency with TrypLE (Thermo Fisher Scientific).

Instrument

Canto II analyzer (BD Biosciences)

Software

FlowJo (v10)

Cell population abundance

Purity check was performed on a small fraction of cells (>95%).

Gating strategy

Cells were gated in the following sequence (X and Y axis): 1. Cells (FSC-A and SSC-A); 2. Single cells (SSC-A and SSC-H); 3. Live cells (DAPI and SSC-A). Furthermore, cell-surface markers combinations were used to identify the following population: in-vitro defined HE (CD144+CD34+CD73-CD184 cells at day 4) and in-vitro defined HPC (CD34+CD43+ cells at D7).

☒ Tick this box to confirm that a figure exemplifying the gating strategy is provided in the Supplementary Information.
